# CNV Analysis Associates AKNAD1 with Type-2 Diabetes in Jordan Subpopulations

**DOI:** 10.1038/srep13391

**Published:** 2015-08-21

**Authors:** Rana Dajani, Jin Li, Zhi Wei, Joseph T. Glessner, Xiao Chang, Christopher J. Cardinale, Renata Pellegrino, Tiancheng Wang, Nancy Hakooz, Yousef Khader, Amina Sheshani, Duaa Zandaki, Hakon Hakonarson

**Affiliations:** 1 Department of Biology and Biotechnology, Hashemite University, Zarqa, Jordan; 2 Cell Therapy Center, University of Jordan, Amman, Jordan; 3 Center for Applied Genomics, Abramson Research Center, Children’s Hospital of Philadelphia, Philadelphia, PA 19104, USA; 4 Department of Computer Science, New Jersey Institute of Technology, Newark, NJ 07102, USA; 5 Department of Biopharmaceutics and Clinical Pharmacy Faculty of Pharmacy-University of Jordan, Amman, Jordan; 6 Faculty of pharmacy, Zarqa University, Zarqa, Jordan; 7 Department of Community Medicine, Public Health and Family Medicine, Faculty of Medicine, Jordan University for Science and Technology, Irbid, Jordan; 8 Division of Human Genetics, The Children’s Hospital of Philadelphia, Philadelphia, PA 19104, USA; 9 Department of Pediatrics, University of Pennsylvania School of Medicine, Philadelphia, PA 19104, USA

## Abstract

Previous studies have identified a number of single nucleotide polymorphisms (SNPs) associated with type-2 diabetes (T2D), but copy number variation (CNV) association has rarely been addressed, especially in populations from Jordan. To investigate CNV associations for T2D in populations in Jordan, we conducted a CNV analysis based on intensity data from genome-wide SNP array, including 34 T2D cases and 110 healthy controls of Chechen ethnicity, as well as 34 T2D cases and 106 healthy controls of Circassian ethnicity. We found a CNV region in protein tyrosine phosphatase receptor type D (*PTPRD*) with significant association with T2D. *PTPRD* has been reported to be associated with T2D in genome-wide association studies (GWAS). We additionally identified 16 CNV regions associated with T2D which overlapped with gene exons. Of particular interest, a CNV region in the gene AKNA Domain Containing 1 (*AKNAD1*) surpassed the experiment-wide significance threshold. Endoplasmic reticulum (ER)-related pathways were significantly enriched among genes which are predicted to be functionally associated with human or mouse homologues of *AKNAD1*. This is the first CNV analysis of a complex disease in populations of Jordan. We identified and experimentally validated a significant CNVR in gene *AKNAD1* associated with T2D.

The chronic disease diabetes has a prevalence of 347 million people worldwide^1^, and this number is constantly increasing. It is estimated that by year 2030, diabetes will become the 7^th^ leading cause of death^2^. In Jordan, 16% of citizens over the age of 18 have diabetes and another 23.8% are likely to develop diabetes, according to a recent survey^3^. Among diabetic cases world-wide, 90% are classified as type 2 diabetes (T2D) which is characterized by impaired insulin secretion from the beta cells of the pancreas and defective insulin action in adipose tissue and other body organs^4^.

Over the past decade genome wide association studies (GWAS) have been fruitful in linking regions of the human genome with T2D and related metabolic traits, identifying and replicating nearly 100 susceptibility loci^5^,^6^,^7^,^8^. However, these GWAS findings have significantly underestimated the heritability of T2D^5^, the so-called missing heritability problem. In addition, most of these T2D GWAS have been performed in Northern Europeans, although some have been done in African^9^ and Asian^10^,^11^ populations.

Copy number variants (CNVs) account for a major proportion of human genetic variation and have been expected to complement SNPs in implicating genetic susceptibility loci for common diseases. The examination of CNVs may help to unravel the unknown genetic architecture of T2D. For example, recurrent CNVs like deletions at 16p11.2 were found in 0.7% of morbid obesity cases in genetic analyses of several European cohorts^6^. Several studies demonstrated that large and rare CNVs collectively associate with extreme early-onset obesity^12^ and variation in body mass index^13^.

In this study, we investigated the potential role of rare variants in T2D, by performing CNV analysis on two ethnic populations of ancient descent, the Circassians and the Chechens, which were genotyped by Illumina single nucleotide polymorphism (SNP) arrays. The Circassians and the Chechens are the largest indigenous nationalities of the North Caucasus^14^, which descend from a single ancient origin with later divisions along linguistic and geographic borders^15^. A large diaspora of Circassians were relocated to Jordan and other regions of the Ottoman Empire as a result of war with the Russian Empire in 1864^16^. These immigrants choose to be endogamous and have kept a distinct sense of identity and ethnicity during their last one hundred and fifty years of residence in Jordan^17^. Therefore, the Circassian and Chechen communities in Jordan are genetically different from the Arab population and represent unique populations for genetic study. The epidemiology of diabetes in the Circassian and Chechen communities in Jordan has been studied, showing a prevalence of impaired fasting glycemic control to be 18.5% for Circassians and 14.6% for Chechens while the prevalence of diabetes was reported to be 9.6% for Circassians and 10.1% for Chechens^18^.

## Results

**Figure 1:**
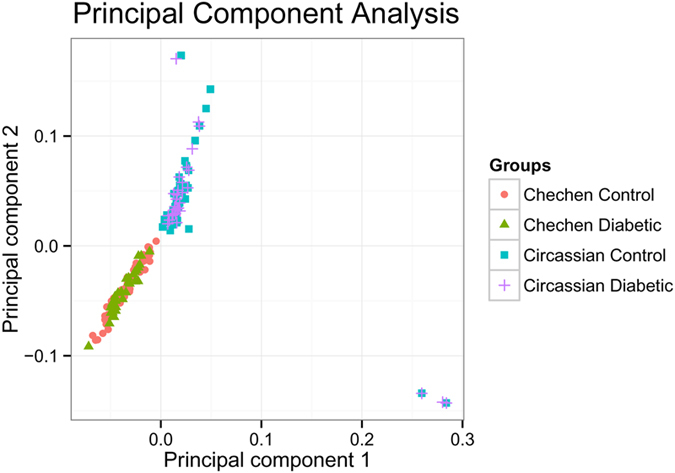
Principal component analysis of the combined cohort. High overlap between diabetic cases and controls are observed within each ethnic group.

To study the potential CNV association with T2D in Jordan, we recruited 284 participants. Specifically, for the Chechen population, we have 34 cases and 110 controls, of which 60 are males and 84 are females. For Circassians, we have 34 cases and 106 controls, of which 61 are males and 79 are females. After quality control (QC) of genotyping data, 208 samples were retained in the analysis. By principal component analysis (PCA), we successfully separate the Chechen and the Circassian ethnic groups and we observed the high overlap between cases and controls within each ethnic group on the PCA plot (Fig. 1). The sample information after QC is summarized in Supplementary Table 1. To boost power in statistical analysis, as well as because of their common ancient origin and distinct population structure from other ethnic groups by continent, we combined the data from these two ethnic groups for further analysis.

The characteristics of CNVs called in cases and controls after QC are shown in Table 1. An average number of 75.2 CNVs including 20.0 deletions and 55.2 duplications were called for each case. For the controls, we detected 14.0 deletions and 50.1 duplications per individual which results in a total of 64.1 CNVs per individual. The average number of deletions each case has is slightly more than that of each control.

To identify CNVs associated with T2D in an unbiased manner, we adopted a segment-based scoring approach^19^. By using this approach, we conducted an unbiased analysis in search for consecutive probes with copy number variation enriched in cases compared to controls. CNV statistics analyzed from genome-wide SNP array are shown in Supplementary Figure 1.

Previous candidate gene studies and GWAS focusing on SNP association have yielded a number of genes showing nominally or even genome-wide significant associations with T2D. We checked their association with T2D in our study and found Protein Tyrosine Phosphatase, Receptor Type, D (*PTPRD*) overlaps with a CNVR significantly associated with T2D (Table 2). This CNVR, chr9:10063148-10070622, falls into introns of the *PTPRD* gene.

**Figure 2:**
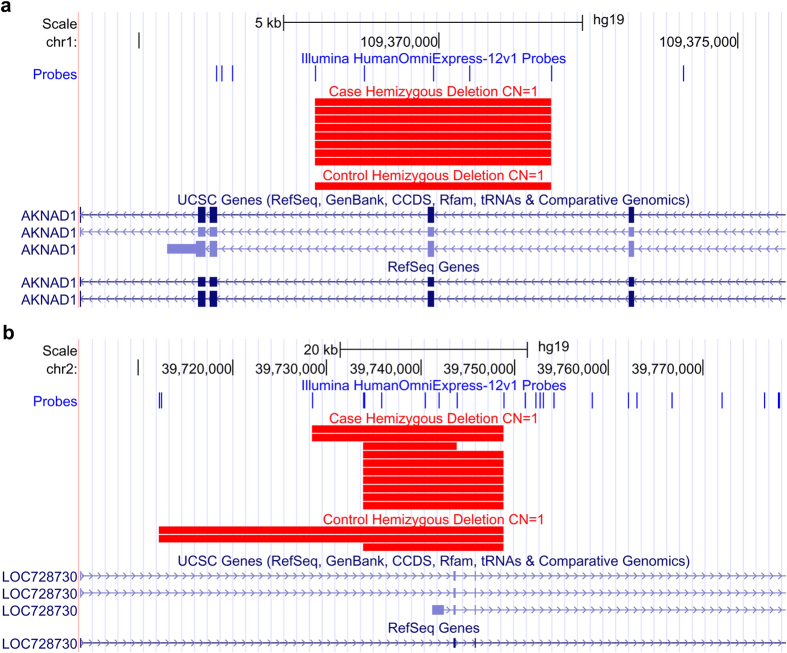
CNVRs chr1:109,367,944–109,371,874 (**a**) and chr2:39,733,850–39,748,858 (**b**) enriched in diabetes cases. Red rectangles represent deletions in cases and controls.

Among the nominally significant loci, there are 16 regions that overlap with gene exons (Table 3) which are very likely to have direct impact on the gene products. Taking into account of upfront SNP QC for CNV analysis and SNP collapsing into CNVR, we defined the experiment-wide significance level of *P *< 5 × 10^−4^ (Methods). Among the 16 regions, CNVR chr1:109367944-109371874 reached the such significance level and with only 1 control sample containing a deletion in this region (Table 3 and Fig. 2a). This region overlaps with one exon and two introns of the gene AKNA Domain Containing 1 (*AKNAD1*, *C1orf62*), containing an AKNA domain which is present in AT-hook-containing transcription factors. Another CNVR of *P *< 5 × 10^−4^ is located at chr2:39733850-39748858 (Table 3 and Fig. 2b). Both these CNVRs are deletions.

**Figure 3:**
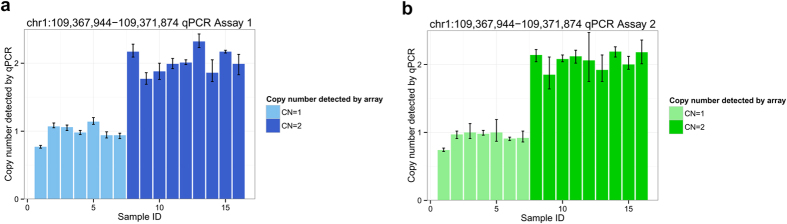
qPCR validation of CNVR chr1:109,367,944–109,371,874. Two independent probes were used, results of which are shown in (**a**) and (**b**) respectively. Copy number (CN) was calculated from triplicate runs per probe for each sample by using CopyCaller (Applied Biosystems). The copy number range bars indicate the minimal and maximal copy number calculated for each sample. Diabetic samples carrying the CNVR detected by SNP array are shown in light-blue in (**a**) or light-green in (**b**). Other samples with CN = 2 at this locus were randomly selected from our cohort, which are represented by dark-blue in (**a**) or dark-green in (**b**). The last sample of CN = 2 in each panel represents a sample of mixed human genomic DNA from Promega.

We further tested the top CNVR of chr1:109367944-109371874 with quantitative polymerase chain reaction (qPCR) an independent experimental assay (Fig. 3). All diabetic samples carrying a CNV at this locus except one sample that was unavailable for analysis were included in the experiment. Eight randomly selected samples without CNVs detected on the array were used as copy number controls (CN = 2). A sample of mixed human genomic DNA from Promega was also included as a control. Our qPCR experiment yielded consistent results to those detected on the Illumina array.

We examined the reported expression levels of *AKNAD1*^20^ in several tissues which are key to diabetes^21^, including adipocyte, liver, pancreas, pancreatic islet, skeletal muscle and smooth muscle (Supplementary Figure 2). *AKNAD1* showed a uniformly low expression level in these tissues, which is similar to several other genes in T2D associated susceptibility loci, such as *MTNR1B*^22^,^23^,^24^, *ADAMTS9*^25^, *THADA*^25^. The correlations of expression pattern between *AKNAD1* and *ADAMTS9*, *THADA* are over 0.9^21^,^26^.

To expand our understanding of the biological function of these CNVs, we conducted functional association network analysis and pathway analysis. By using STRING 9.1^27^,^28^ based on different knowledge sources, we detected 11 genes having predicted protein-protein interactions with human AKNAD1 (Supplementary Figure 3). Microtubule-based movement and processes along with other microtubule-related pathways were significantly enriched among these genes (Supplementary Table 2). Using another approach, FunCoup 3.0^29^,^30^, we similarly found 17 genes which are of predicted functional association with murine *AKNAD1* (Supplementary Figure 4). Interestingly, endoplasmic reticulum (ER) related pathways were significantly enriched among these genes (Supplementary Table 3). ER stress has been known to be a major cause of beta cell death and T2D. Interestingly, when we used the 53 gene names listed in Table 3 as input for STRING search (Supplementary Figure 5), pathways of pancreatic cancer, which has been shown to be positively associated with diabetes^29^, and the pathway of type 2 diabetes showed evidence of nominal significance (Supplementary Table 4). The identification of these pathways suggested the validity of our study. Furthermore, a similar network analysis in FunCoup 3.0 (Supplementary Figure 6) yielded murine homologues of three genes (*MAPK8IP3*, *RTN2*, *MMEL1*), and two additional functional partners (*Vapa* and *Ddost*) in ER-related pathway (Supplementary Table 5).

CNVR chr1:109367944-109371874 covers not only an exon but also the flanking introns of the gene *AKNAD1*. Several sources of data suggested that this region may also contain transcriptional regulation-associated chromatin modifications. By Epigenomics Roadmap^30^ histone modifications, we observed peaks of histone markers in the intron regions covered by the CNVR in pancreatic islets (Supplementary Figure 7a) and liver cells (Supplementary Figure 7b), such as H3K27me3 which is associated with transcriptional repression^31^, H3K36me3, H3K4me1, H3K4me3 and H3K27Ac which usually correlate with transcriptional activation^32^,^33^,^34^,^35^. DNaseI hypersensitivity signals in this region have also been detected in pancreatic islets (Supplementary Figure 7c) based on ENCODE^36^ Open Chromatin by DNaseI HS and FAIRE Tracks. In another two genome-wide epigenetic studies in human pancreatic islets, this region was also predicted to contain enhancer regions^37^ (Supplementary Figure 7d) or open chromatin region bound by CTCF (chr1: 109,370,419–109,371,565)^38^, likely depending on the different experimental methods or computational algorithms used. By Haploreg^39^ search, we further found that this region also contains SNPs detected to be protein-bound in diabetes relevant cell lines of the ENCODE^36^ project or have been predicted to alter transcription factor binding motifs (Supplementary Table 6). We noted that 5 SNPs in this region are found to have CTCF bound by Chromatin immunoprecipitation (ChIP) in muscle, pancreatic and liver cell lines, which is consistent with the observation in human pancreatic islets^38^.

## Discussion

In this study, we conducted genome-wide SNP array based CNV association analysis in two ethnic groups in Jordan, leading us to identify and experimentally validate a significant CNVR in the gene *AKNAD1*. This is the first CNV analysis of a complex disease for populations in Jordan.

Though T2D has been well studied by GWAS, especially among European populations, with the fruitful yield of ~100 susceptibility loci^6^,^7^,^8^, the association between CNV and T2D has rarely been investigated and no such study has been carried out in Chechen and Circassian populations. Previous studies have reported ethnic differentiation of CNVs^40^,^41^,^42^ and it has been commonly recognized that genes, environment, and their interactions together shape complex disease.

The experiment-wide significant CNVR chr1:109367944-109371874 is a deletion region and covers one exon and two introns of gene *AKNAD1*. This gene contains an AKNA domain, whose biological function is uncharacterized. Our protein-protein interaction network search detected its predicted interactions with a group of kinesin family members and microtubule associated proteins, which resulted in the highly significant enrichment of GO biological processes of “microtubule-based movement” and “microtubule-based process.”

The mouse homolog of *AKNAD1* has not been well characterized, but similar functional interaction network searches also yield a group of predicted functional partners enriched in endoplasmic reticulum related processes. Although there is no overlap between the group of predicted human *AKNAD1* interactors and those of mouse *AKnad1*, the biological pathways enriched are highly related. The ER is a cellular organelle which is composed of interconnected networks of tubular membranes^43^,^44^. Terasaki *et al.* has pointed out the interdependence between ER and microtubules based on their experimental observations: the co-occurrence of microtubule polymerization and ER extension combined with ER retraction due to long-term microtubule depolymerization^45^. Further accumulated evidence led to the conclusion that microtubules are necessary for ER localization and dynamics^46^,^47^. Later studies also indicated that the microtubule motor kinesin mediates membrane traffic between Golgi and ER^46^,^48^,^49^.

ER stress has been recognized as one of the important factors in the onset and progression of T2D (reviewed by^50^,^51^,^52^). T2D is characterized by insulin resistance and beta cell dysfunction^53^. The pancreatic beta cells are the only source of insulin, which functions to reduce blood glucose levels^54^,^55^,^56^ and the ER is the location of proinsulin synthesis and processing. In response to changes in blood glucose level, and other physiological and environmental fluctuations, the protein folding load in the ER can vary dramatically. Therefore, maintaining the homeostasis of the ER is critical for the normal function of beta cells. When protein overload occurs or the ER milieu is compromised, the unfolded protein response (UPR) is activated, which is an adaptive response mechanism to restore normal function and establish its homeostasis again (reviewed by^57^). However, in situations of severe ER stress, UPR triggers apoptotic pathways (reviewed by^50^). Various types of nutrient toxicity can induce ER stress such as lipotoxicity, glucotoxicity^58^ and islet amyloid^59^. Apoptotic signaling triggered by severe and prolonged ER stress results in beta cell dysfunction, cell death and eventually T2D (reviewed by^50^). ER stress can also activate the inflammatory response and production of cytokines which also lead to beta-cell death and T2D^60^.

Interestingly, one of the predicted human *AKNAD1* interactors, *DEAF1* (deformed epidermal autoregulatory factor 1) has been reported to be involved in the pathogenesis of type 1 diabetes (T1D)^61^,^62^,^63^,^64^. Alternative splicing of DEAF1 inhibits the transcription of peripheral tissue antigens^62^,^63^ as well as suppresses gene translation by downregulating the transcription of translation initiation factor gamma 3 (Eif4g3) in lymph nodes^64^. Therefore, DEAF1 plays a role in destruction of peripheral tolerance which leads to T1D^62^. Evidence suggests that T1D and T2D are different but related diseases^65^,^66^.

Exon deletion can have detrimental effects on gene transcription and translation, which usually results in dysfunctional protein products, even though exon skipping can occur. CNVR chr1:109367944-109371874 covers not only an exon but also a large part of its flanking introns, where pancreatic islet and liver specific transcriptional regulatory elements may reside as shown in Supplementary Figure 7 and Supplementary Table 6. Thus, the expression level and the biological function of the transcripts are likely to be severely affected. Intriguingly, three SNPs in the gene-rich region where *AKNAD1* is located were associated with subcutaneous adipose tissue distribution in HIV-infected men^67^, while adipose tissue distribution is often altered in patients with T2D^68^. In conclusion, the biological function of *AKNAD1* merits further study, especially in the context of T2D.

While we made novel discovery of the association between *AKNAD1* CNVR and T2D among Jordan subpopulations, there are certain limitations in our study. First, our sample size is small, therefore some associations may be missed due to limited power. Another limitation is the potential confounding from population structure. As the two Jordanian subpopulations are descended from a single ancient origin, and have a distinct population structure compared to other ethnic groups by continent, we combined them in our study. There is an almost equal number of cases from each subpopulation, therefore we do not expect confounding due to this combination. However, we could not fully exclude the possibility of within-population confounding. Because our sample size is small and the CNVR is of low frequency, there is no good consensus way in the CNV field how to address this potential issue. Therefore, cohorts of larger sample size and biological functional studies are needed to further examine the role of this association in T2D etiology.

## Material and Methods

### Ethics Statement

The study has been approved by the institutional review board committee at the National Center for Diabetes, Endocrinology and Genetics of Jordan; and the methods were carried out in accordance with the approved guidelines. The written informed consent was signed and obtained from all participants.

### Research Subjects

A total of 144 subjects including cases and controls from the Chechen population in Jordan and 140 subjects from the Circassian population in Jordan were recruited, with signed consent of agreeing to participate. A survey was completed by each participant and pedigree information was collected. Any Chechen subject whose parents, grandparents or great grandparents from maternal or paternal side are of non-Chechen heritage was excluded from the study. Similar exclusion criteria were applied to the Circassian population.

### Phenotype confirmation

In our study, cases with diabetes mellitus were defined according to the following criteria: Diagnosis of diabetes was known to the patient or, if fasting serum glucose is equal to or greater than 7 mmol/l (126 mg/dl) based on the ADA definition. Impaired fasting glucose was defined as a fasting serum glucose level of between 6.1 mmol/L (100 mg/dl) and 7 mmol/l. HbA1c was used to evaluate glycemic control. Any previously diagnosed diabetic patient with HbA1c >7% was classified as having ‘unsatisfactory’ glycemic control.

### Sample collection and SNP genotyping

Nine ml of blood sample was collected from each participant and genomic DNA was extracted by using the phenol-chloroform protocol. High-throughput, genome-wide SNP genotyping was conducted using the Infinium II OMNI-Express BeadChip technology (Illumina), at the Center for Applied Genomics (CAG) at the Children’s Hospital of Philadelphia (CHOP), USA, according to manufacturer’s standard protocol. Both SNP genotype data and intensity data including information of Log R Ratio (LRR) and B Allele Frequency (BAF) was extracted from GenomeStudio project files.

### CNV calling

We generated CNV calls using PennCNV^69^ which is based on a trained hidden Markov model and combines information of LRR and BAF for each SNP marker, as well as population frequency of the B allele for CNV calling.

### CNV Quality Control

As described in previous publication^70^, we evaluated the CNV quality metrics which include sample call rate, cryptic relatedness between samples, the standard deviation of LRR (LRR SD) as a measure of intensity noise, |GCWF| as a measure of intensity waviness, and the number of CNVs per sample. Based on the distribution of each measure of the quality metrics, outlier samples of low quality were excluded from further analysis. Only samples with genotyping rate >98%, LRR SD <0.3 or |GCWF| <0.02 and CNV count <100 were included. Furthermore, genome-wide identity-by-descent analysis was carried out using PLINK^71^ and duplicated or related samples which of identity-by-descent score (PI_HAT) >0.30 were detected. One sample from each pair of such cryptic related samples was removed.

### Principal Component Analysis

After QC, we performed PCA based on SNP genotypes of a set of LD (Linkage disequilibrium)-pruned SNPs using software EIGENSTRAT^72^.

### CNV association analysis

We tested CNV association with T2D case control status via ParseCNV which uses an unbiased segment-based scoring approach to identify CNV regions (CNVRs) associated with the disease status^19^. Briefly, for each SNP, CNV frequency between cases and controls were compared by Fisher exact test. Then neighboring SNPs were collapsed into CNVRs which constitute genomic span of consecutive probes in proximity (<1 MB) and having comparable significance (+/−1 log p-value) in Fisher exact test when comparing case to control status. The local lowest SNP *P*-value in Fisher exact test was used to represent the association of CNVR with disease status. We also evaluated the proper experiment-wide multiple testing correction in our study. Among the total of 733,202 SNPs on the Infinium II OMNI-Express BeadChip, 168 probes (0.023%) showed deletion and 851 (0.12%) showed duplication in at least three or more unrelated cases in our cohort (frequency ≥4.76%). We chose this cutoff because it is the minimal case frequency that could yield nominal statistical significance and reproducibility for a CNV in a given region in Fisher exact test when comparing to controls. This upfront SNP filtering is analogous to 1% minor allele frequency selection in genome-wide association studies on SNP genotype data. Afterwards, such SNPs were collapsed into 20 deletion CNVRs and 69 duplication CNVRs. Therefore, we conducted 89 tests, resulting in an experiment-wide significance threshold of *P* = 5.62 × 10^−4^ with multiple testing correction, which is similar to what we encountered in other CNV analyses based on genome-wide SNP arrays, taking into account of upfront SNP QC for CNV analysis and SNP collapsing into CNVR^70^ (http://parsecnv.sourceforge.net/). However, this experiment-wide significance threshold is higher than the conventional significance cutoff of *P *= 5 × 10^−8^ for a GWAS with SNP genotypes. Annotation of each CNVR was also generated by ParseCNV.

### CNV validation

Quantitative polymerase chain reaction (qPCR) was used to validate selected CNVRs. TaqMan copy number probes Hs03345652_cn or Hs00909430_cn from Applied Biosystems (Carlsbad, Calif.) were used in qPCR reactions, following the manufacturer’s standard protocol.

### Gene expression analysis

The expression profiles of genes examined and their correlations were downloaded from the BIOGPS^21^,^26^ website http://biogps.org. The bargraph with standard deviation of tissue specific expression of each gene was generated using statistical and graphics software R^73^.

### Network and pathway analysis

*AKNAD1* or all 53 genes shown in Table 3 were used as input for software STRING 9.1^27^,^28^ to search for known or predicted protein-protein interactions between query genes and additional functional partners. To reduce network complexity, when 53 genes were entered, only 10 more partners of strongest interaction evidence were added to the network of query genes. Similar searches were also performed using software FunCoup 3.0^74^,^75^ for murine homologues of *AKNAD1* and all 53 genes shown in Table 3.

## Additional Information

**How to cite this article**: Dajani, R. *et al.* CNV Analysis Associates AKNAD1 with Type-2 Diabetes in Jordan Subpopulations. *Sci. Rep.* **5**, 13391; doi: 10.1038/srep13391 (2015).

## Supplementary Material

Supplementary Information

## Figures and Tables

**Table 1:** Profiles of CNVs called in the cases and controls after QC.

	Cases (N=63)	Controls (N=145)	Comparison *P*
**Total number of CNVs**	4736	9303	
Deletions	1258	2037	
Duplications	3478	7266	
**Average number of CNVs per subject**	75.2	64.1	0.12
Deletions	20.0	14.0	0.029
Duplications	55.2	50.1	0.51

Comparison *P* = *P*-value for comparison between cases and controls via *t*-test.

**Table 2:** CNVRs significantly enriched in T2D cases and overlap with previously identified T2D associated genes.

CNVR(hg19)	P	Cases CNV	Controls CNV	Gene	CNV Type	Cytoband
chr9:10063148-10070622	0.00065	6	0	PTPRD	Dup	9p23

Dup = Duplication; Cases CNV = the number of cases having CNV in this region; Controls CNV=the number of controls having CNV in this region.

**Table 3:** CNVRs significantly enriched in T2D cases and overlap with gene exons.

CNVR(hg19)	*P*	Cases CNV	Controls CNV	Gene	CNV Type	Cytoband
**chr1:109367944-109371874**	**0.000347**	**8**	**1**	**AKNAD1(C1orf62)**	**Del**	**1p13.3**
**chr2:39733850-39748858**	**0.000471**	**10**	**3**	**LOC728730 (AK090796,AK095178,BC042073)**	**Del**	**2p22.1**
chr3:162626585-162640469	0.00065	6	0	BC073807	Dup	3q26.1
chr2:242926381-243034519	0.00139	9	3	FLJ38379,LOC441309	Del	2q37.3
chr1:200974641-200975064	0.00686	13	10	KIAA0449,KIF21B	Del	1q32.1
chr11:23690735-24551109	0.00786	4	0	DKFZp451K1618,LUZP2	Dup	11p14.3
chr16:1183677-1807896	0.00851	8	4	BAIAP3,BC061641,BC114455,C16orf38,C16orf42,C16orf91,CACNA1H,CLCN7,CRAMP1L,DKFZp434O1216,GNPTG,HN1L,IFT140,KIAA1066,KIAA1426,MAPK8IP3,TELO2,TMEM204,TPSAB1,TPSB2,TPSD1,TPSG1,UBE2I,UNKL	Del	16p13.3
chr1:2538209-2587026	0.0104	5	1	MMEL1	Dup	1p36.32
chr1:25598275-25642595	0.0122	9	5	AX747205,RHD	Del	1p36.11
chr9:96015256-96022116	0.0238	6	3	WNK2	Del	9q22.31
chr1:186307064-186372865	0.0238	6	3	C1orf27,OCLM,TPR	Dup	1q31.1
chr21:23345922-23391186	0.0269	3	0	BC039377	Dup	21q21.1
chr10:106026539-106032212	0.0269	3	0	GSTO1,GSTO2	Dup	10q25.1
chr12:56502925-56520375	0.0269	3	0	PA2G4,RPL41,ZC3H10	Dup	12q13.2
chr19:45997347-46002456	0.0269	3	0	FLJ40125,RTN2	Dup	19q13.32
chr6:31793716-31803745	0.0302	4	1	C6orf48,HSPA1B,SNORD48	Dup	6p21.32

The highlighted ones reached experiment-wide significance level of *P* < 5 × 10^−4^. Del = Deletion; Dup = Duplication; Cases CNV = the number of cases having CNV in this region; Controls CNV = the number of controls having CNV in this region.
