# Supplementary material for: CNV Analysis Associates AKNAD1 with Type-2 Diabetes in Jordan Subpopulations
Source: Sci Rep. 2015 Aug 21;5:13391. doi: 10.1038/srep13391 (PMC4543987; doi:10.1038/srep13391)
Supplement: Supplementary Information [file srep13391-s1.pdf]

CNV Analysis Associates AKNAD1 with Type-2 Diabetes in Jordan Subpopulations

Rana Dajani, Jin Li, Zhi Wei, Joseph T. Glessner, Xiao Chang, Christopher J. Cardinale, Renata Pellegrino, Tiancheng Wang, Nancy Hakooz, Yousef Khader, Amina Sheshani, Duaa Zandaki, Hakon Hakonarson

Supplementary Information

Supplementary Tables

Supplementary Table 1. Number of T2D-cases vs controls and their ethnic distribution following QC.

Ethnicity	control	case	Total
**Chechen**	86	33	119
**Circassian**	59	30	89
**Total**	145	63	**208**

Supplementary Table 2. Significantly enriched pathway terms among genes which are of predicted protein-protein interaction with human *AKNAD1* by using STRING 9.1 [1,2].

**GO biological process**				
GO_id	Term	NumberOfGenes	*P*-value	*P*-value_fdr
GO:0007018	microtubule-based movement	5	1.57×10^{-8}	1.94×10^{-4}
GO:0007017	microtubule-based process	6	3.99×10^{-8}	2.47×10^{-4}
**GO molecular function**				
GO_id	Term	NumberOfGenes	*P*-value	*P*-value_fdr
GO:0008017	microtubule binding	6	1.34×10^{-10}	4.33×10^{-7}
GO:0003777	microtubule motor activity	5	2.23×10^{-10}	4.33×10^{-7}
GO:0015631	tubulin binding	6	9.54×10^{-10}	1.23×10^{-6}
GO:0003774	motor activity	5	1.98×10^{-9}	1.92×10^{-6}
GO:0008092	cytoskeletal protein binding	6	8.30×10^{-7}	6.44×10^{-4}
GO:0032403	protein complex binding	6	1.05×10^{-6}	6.81×10^{-4}
GO:0017111	nucleoside-triphosphatase activity	5	2.05×10^{-5}	1.04×10^{-2}
GO:0016462	pyrophosphatase activity	5	2.61×10^{-5}	1.04×10^{-2}
**GO cellular compartment**				
GO_id	Term	NumberOfGenes	*P*-value	*P*-value_fdr
GO:0005871	kinesin complex	5	3.53×10^{-11}	5.11×10^{-8}
GO:0005875	microtubule associated complex	5	2.65×10^{-9}	1.92×10^{-6}
GO:0005874	microtubule	6	1.36×10^{-8}	6.55×10^{-6}
GO:0044430	cytoskeletal part	6	2.01×10^{-5}	7.28×10^{-3}

P-value_fdr=False discovery rate adjusted *P*-value.

Supplementary Table 3. Significantly enriched pathway terms among genes which are functionally associated with mouse *AKnad1* predicted by FunCoup 3.0 [3,4].

database	Enriched terms	#Genes	q-value
GO cellular component	**endoplasmic reticulum**	9	4.31×10^{-4}
	mitochondrial envelope	6	9.73×10^{-4}
	sarcoplasmic reticulum	2	3.43×10^{-2}
	sarcoplasm	2	3.43×10^{-2}
	mitochondrion	7	3.43×10^{-2}
KEGG metabolic	**Protein processing in endoplasmic reticulum**	3	4.36×10^{-2}
KEGG signaling	Neurotrophin signaling pathway	2	3.90×10^{-2}
	Wnt signaling pathway	2	3.90×10^{-2}
GO biological process	**endoplasmic reticulum calcium ion homeostasis**	2	1.77×10^{-2}

q-value= False discovery rate adjusted *P*-value.

Supplementary Table 4. Top enriched pathway terms among genes which are of predicted protein-protein interaction with genes the exons of which overlap with significant CNVRs by using STRING 9.1 [1,2].

**GO biological process**				
GO_id	Term	NumberOfGenes	*P*-value	*P*-value_fdr
GO:0033365	protein localization to organelle	8	$2.84x10^{-6}$	$3.52x10^{-2}$
GO:0034504	protein localization to nucleus	5	$1.08x10^{-5}$	$6.20x10^{-2}$
GO:0007258	JUN phosphorylation	2	$1.50x10^{-5}$	$6.20x10^{-2}$
GO:0072594	establishment of protein localization to organelle	6	$4.06x10^{-5}$	$8.76x10^{-2}$
GO:0006606	protein import into nucleus	4	$4.96x10^{-5}$	$8.76x10^{-2}$
GO:0044744	protein targeting to nucleus	4	$4.96x10^{-5}$	$8.76x10^{-2}$
GO:0032436	positive regulation of proteasomal ubiquitin-dependent protein catabolic process	3	$6.69x10^{-5}$	$9.20x10^{-2}$
**KEGG pathways**				
KEGG_id	Term	NumberOfGenes	*P*-value	*P*-value_fdr
hsa03013	RNA transport	6	$1.58x10^{-6}$	$3.75x10^{-4}$
hsa05160	Hepatitis C	4	$3.59x10^{-4}$	$3.16x10^{-2}$
hsa05210	Colorectal cancer	3	$4.74x10^{-4}$	$3.16x10^{-2}$
hsa04010	MAPK signaling pathway	5	$5.59x10^{-4}$	$3.16x10^{-2}$
hsa05212	Pancreatic cancer	3	$6.67x10^{-4}$	$3.16x10^{-2}$
hsa05200	Pathways in cancer	5	$1.35x10^{-3}$	$5.32x10^{-2}$
hsa05216	Thyroid cancer	2	$2.64x10^{-3}$	$8.93x10^{-2}$
hsa04722	Neurotrophin signaling pathway	3	$4.28x10^{-3}$	0.127
hsa04120	Ubiquitin mediated proteolysis	3	$5.45x10^{-3}$	0.144
hsa04930	Type II diabetes mellitus	2	$6.72x10^{-3}$	0.155
hsa04310	Wnt signaling pathway	3	$7.21x10^{-3}$	0.155
hsa00480	Glutathione metabolism	2	$8.25x10^{-3}$	0.163
hsa04621	NOD-like receptor signaling pathway	2	$1.03x10^{-2}$	0.180
hsa05131	Shigellosis	2	$1.06x10^{-2}$	0.180
hsa05120	Epithelial cell signaling in Helicobacter pylori infection	2	$1.37x10^{-2}$	0.196
hsa04920	Adipocytokine signaling pathway	2	$1.41x10^{-2}$	0.196
hsa00980	Metabolism of xenobiotics by cytochrome P450	2	$1.49x10^{-2}$	0.196
hsa04622	RIG-I-like receptor signaling pathway	2	$1.53x10^{-2}$	0.196
hsa00982	Drug metabolism - cytochrome P450	2	$1.57x10^{-2}$	0.196

P-value_fdr=False discovery rate adjusted *P*-value.

Supplementary Table 5. Top enriched pathway terms among genes which are functionally associated with murine homologues of genes the exons of which overlap with significant CNVRs, by using FunCoup 3.0 [3,4].

Database	Enriched terms	#Genes	q-value
Go cellular component	chromosome	6	0.138
	nuclear lumen	8	0.138
	microtubule cytoskeleton	5	0.138
	nucleoplasm	5	0.148
	nucleoplasm part	4	0.213
	**endoplasmic reticulum**	5	0.239

q-value= False discovery rate adjusted *P*-value.

Supplementary Table 6. Functional regulatory elements in the significant CNVR (chr1:109,367,944-109,371,874) of *AKNAD1* found by Haploreg [5] search**.**

chr	pos (hg19)	variant	GERP	SiPhy	Proteins bound	Motifs changed	RefSeq gene	Function annotation
1	109367944	rs10857968				CEBPB,HLF,Hsf,Mef2	AKNAD1	intronic
1	109367979	rs143367096				ATF3,Isl2,Mrg,Pax-4	AKNAD1	intronic
1	109368003	rs146713389	Yes	Yes		Isl2,Nkx2	AKNAD1	intronic
1	109368024	rs191386505	Yes			CCNT2,GATA,Hltf,PU.1	AKNAD1	intronic
1	109368042	rs140290623				CDP,GATA,HDAC2,Pou3f2	AKNAD1	intronic
1	109368052	rs80238007				GCM,Pax-8	AKNAD1	intronic
1	109368112	rs61797358				Irf,STAT	AKNAD1	intronic
1	109368122	rs12759838				Hand1,PLAG1,p300	AKNAD1	intronic
1	109368164	rs115846560				Nanog,Rhox11,Sox	AKNAD1	intronic
1	109368241	rs1338527				GR,MZF1::1-4,NF-Y,SP1,ZBTB7A	AKNAD1	intronic
1	109368249	rs1338526				GR	AKNAD1	intronic
1	109368310	rs183046520				Evi-1	AKNAD1	intronic
1	109368333	rs1338525				CEBPB,Pou2f2,Pou5f1	AKNAD1	intronic
1	109368342	rs188480884				Ik-1,Pou2f2	AKNAD1	intronic
1	109368345	rs192950072				Ik-1,Ik-2,Ik-3,STAT	AKNAD1	intronic
1	109368404	rs1338524				Mrg1::Hoxa9,TATA	AKNAD1	intronic
1	109368453	rs113698066				Cdx2,DMRT2,Foxp3,HNF6,Hoxa10,Hoxb9,Hoxc9,RORalpha1	AKNAD1	intronic
1	109368495	rs184780343				CDP,CEBPG,Foxo,Mef2,ZEB1	AKNAD1	intronic
1	109368512	rs61797359					AKNAD1	intronic
1	109368576	rs200273991				Hand1,Irf,SIX5,Znf143	AKNAD1	intronic
1	109368684	rs187842376				NF-kappaB,STAT	AKNAD1	intronic
1	109368701	rs145336427				E2A,En-1,Ncx	AKNAD1	intronic
1	109368718	rs11102502				NF-kappaB	AKNAD1	intronic
1	109368762	rs11102503				Barhl1,Bbx,Bsx,Dbx2,En-1,Esx1,Hbp1,Hmx,Hoxb4,Isl2,Msx-1,Nkx2,Nr2f2,Pax7,Pbx-1,Phox2a,Prrx2,Vax2	AKNAD1	intronic
1	109369188	rs137943894				Crx	AKNAD1	intronic
1	109369292	rs192613155				E2A,Myf,PLZF,TAL1,ZEB1	AKNAD1	intronic
1	109369340	rs76071396				Evi-1,Foxd3,Foxj1,Foxp1,HDAC2,Irf,Nkx6-1,OTX,p300	AKNAD1	intronic
1	109369363	rs67732846				BCL,DMRT5,HDAC2,Irf,Mef2,Nanog,RXRA,ZEB1,p300	AKNAD1	intronic
1	109369576	rs183760774				BCL,GCNF,NRSF,YY1,Zic	AKNAD1	intronic
1	109369609	rs11102504				ELF1,HES1,Myc,NRSF	AKNAD1	intronic
1	109369610	rs189326009				E2A,ELF1,Maf,Mxi1,Myc,Myf,NRSF,TAL1	AKNAD1	intronic
1	109369612	rs143013016				ELF1,ERalpha-a,Myc,NRSF	AKNAD1	intronic
1	109369727	rs181115306				PPAR,STAT	AKNAD1	intronic
1	109369791	rs11102505					AKNAD1	intronic

1	109369797	rs11102506				Pax-4	AKNAD1	intronic
1	109369849	rs184775863				CHD2,E2F,SP1	AKNAD1	synonymous
1	109369915	rs7551421	Yes			AP-1,Myc	AKNAD1	missense
1	109369939	rs190125386					AKNAD1	intronic
1	109369942	rs181784849					AKNAD1	intronic
1	109370019	rs185999151				Evi-1,Hoxd10,Mef2	AKNAD1	intronic
1	109370156	rs190805172					AKNAD1	intronic
1	109370361	rs7554124				BDP1,CTCF,E2F,Ets,Irf,Rad21,SMC3,SP1,Znf143	AKNAD1	intronic
1	109370390	rs7517916					AKNAD1	intronic
1	109370514	rs7518022				Evi-1,GATA	AKNAD1	intronic
1	109370675	rs61799960				ERalpha-a,GCNF,HNF4,LRH1,Pax-6,RORalpha1,RXRA,SF1	AKNAD1	intronic
1	109370778	rs113232769			CTCF (HSMMtube)	CDP,Pbx-1,Pdx1,RORalpha1,XBP-1	AKNAD1	intronic
1	109370789	rs147300732			CTCF (HSMMtube, HepG2)	AIRE,ERalpha-a,Myc,SIX5,Znf143	AKNAD1	intronic
1	109370886	rs139237792			CTCF (HPAF, HSMM, HSMMtube, HepG2), USF1 (HepG2), RAD21 (HepG2)	Arnt,BHLHE40,Myc	AKNAD1	intronic
1	109370906	rs143150082			CTCF (HPAF, HSMM, HSMMtube, HepG2), USF1 (HepG2), RAD21 (HepG2)	Mtf1,Myc,RXRA,Rad21	AKNAD1	intronic
1	109370918	rs182009787	Yes	Yes	CTCF(HPAF, HSMM, HSMMtube, HepG2), USF1(HepG2), RAD21(HepG2)	AP-2,ATF3,CTCFL,CTCF,Lmo2-complex,Myf,RXRA,Rad21,SMC3,TCF12,Zic	AKNAD1	intronic
1	109371198	rs150846434				Foxa,Foxc1,Foxd1,Foxf2,Foxj1,Foxo,HDAC2,Myf,Pax-5,TCF12,p300	AKNAD1	intronic
1	109371214	rs184922276				ERalpha-a,GR,HEY1	AKNAD1	intronic
1	109371246	rs190798809				CCNT2,NRSF,SP1,STAT	AKNAD1	intronic
1	109371732	rs183164822				TCF12	AKNAD1	intronic
1	109371733	rs139267595				Zfp105	AKNAD1	intronic
1	109371785	rs1277227				CEBPB,E4BP4,Gsc,Nanog,Pou2f2,Pou3f2	AKNAD1	intronic
1	109371874	rs11102515				Brachyury,Irf,LBP-1	AKNAD1	intronic

chr=chromosome; pos=position; GERP and SiPhy-omega are mammalian conservation algorithms implemented Haplogreg search. Cell line abbreviations: HSMMtube= skeletal muscle myotubes differentiated from the HSMM cell line; HPAF=Human pancreatic adenocarcinoma cells; HSMM=skeletal muscle myoblasts; HepG2= hepatocellular carcinoma;

Supplementary Figures

Supplementary Figure 1. Manhattan plot for SNP-based CNV statistics of (a) deletions and (b) duplications.

The SNP position on each chromosome is shown on the X-axis; and the –log *P*-value in the copy number association analysis of each SNP is indicated on the Y-axis.

a

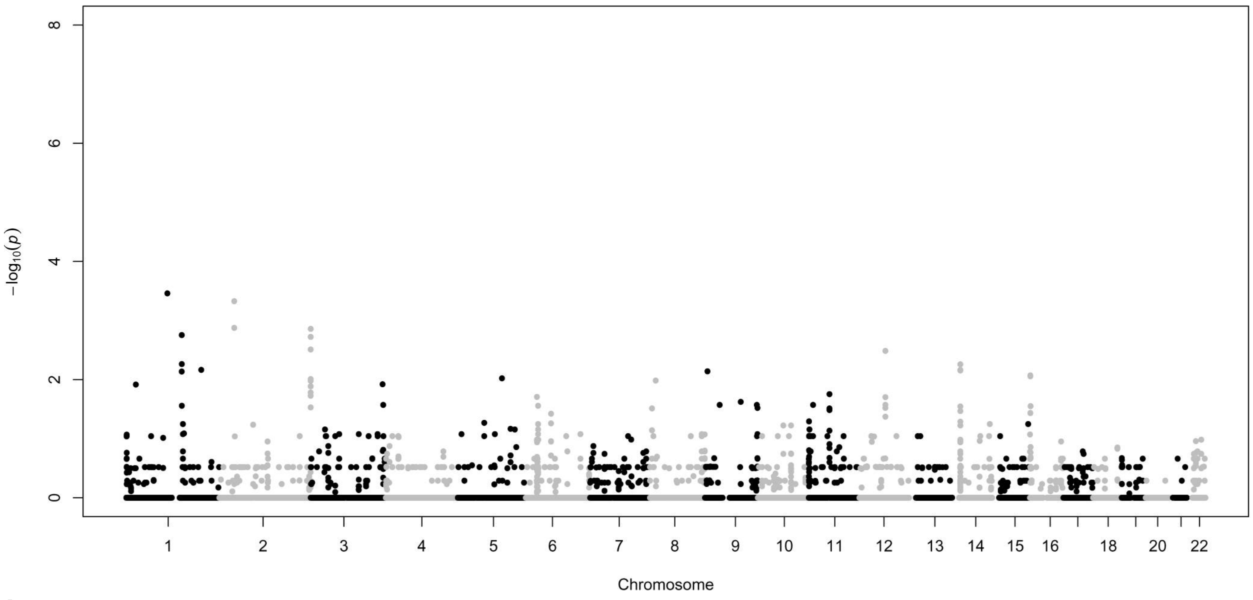

b

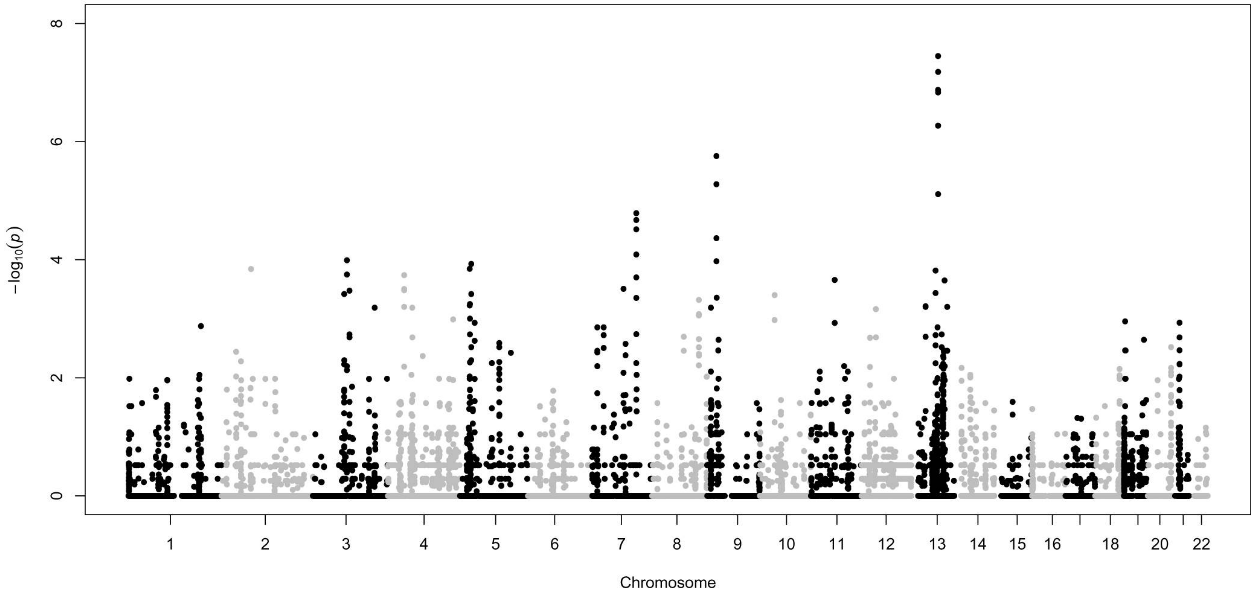

Supplementary Figure 2. The relative expression levels of genes *AKNAD1*, *MTNR1B*, *ADAMTS9* and *THADA* in tissues key to diabetes. The expression profiles of these genes reported in literature[6] were downloaded via the online portal BIOGPS[7,8]. The mean relative expression levels are shown in the bar graph, with standard deviation indicated by the error bars.

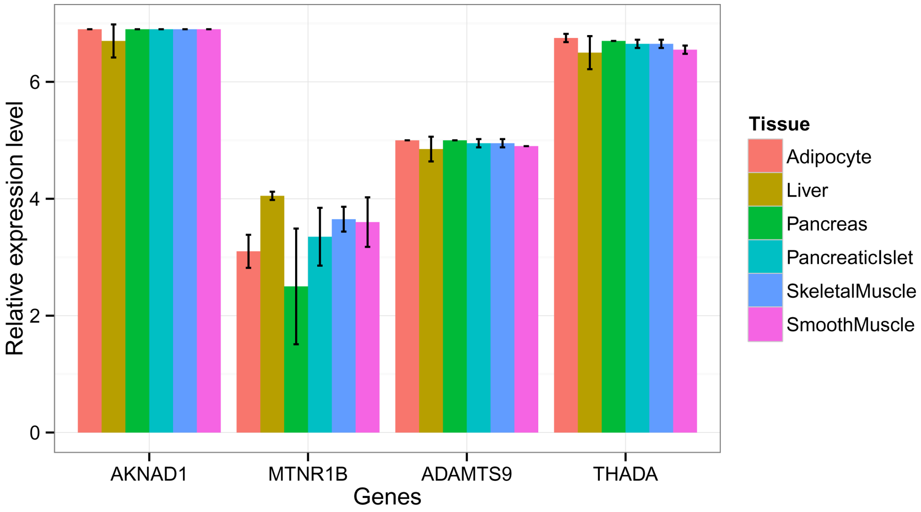

Supplementary Figure 3. Predicted protein-protein interaction network of human AKNAD1. We searched for gene products that are of predicted protein-protein interactions with human AKNAD1 by using STRING 9.1.[1,2] The edges connecting each pair of gene products represent predicted functional links. The prediction methods are based on different sources of evidence which are shown by the edge colors and indicated at the right hand corner.

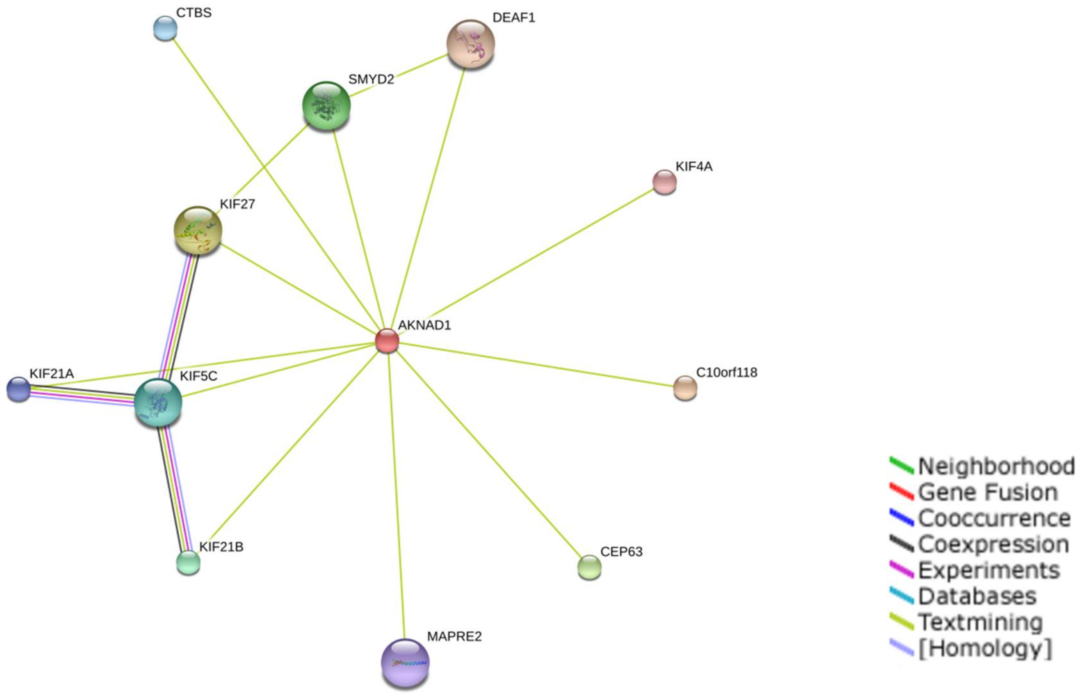

Supplementary Figure 4. Predicted functional coupling network of mouse AKnad1.
We searched for gene products that are functionally associated with AKNAD1 murine homolog AKnad1 by using FunCoup 3.0[3,4].

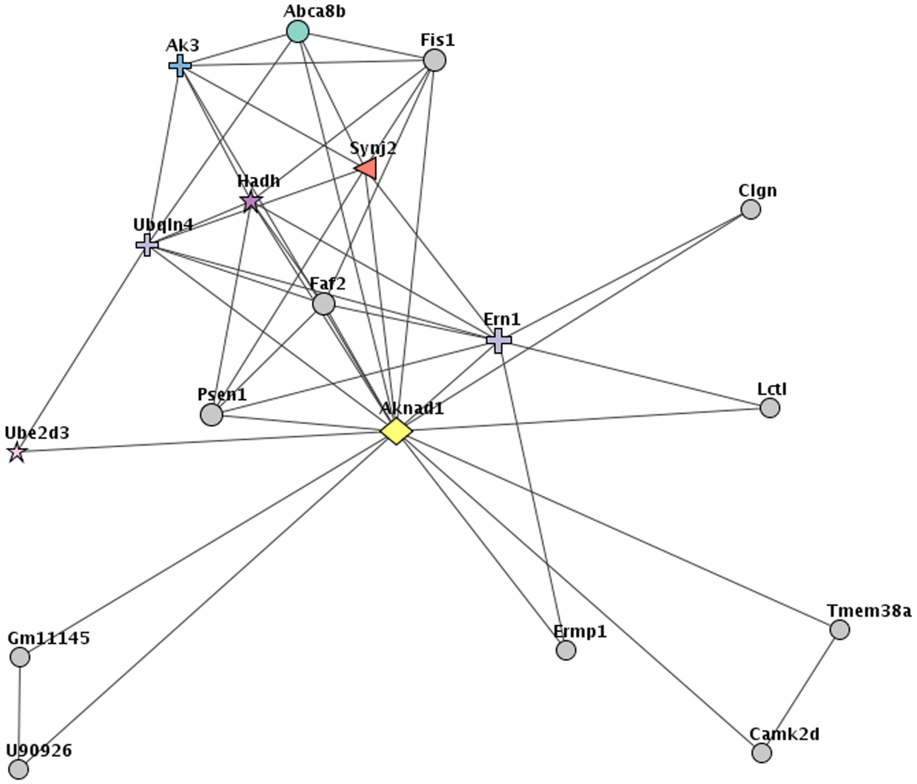

Supplementary Figure 5. Predicted protein-protein interaction network of genes the exons of which overlap with significant CNVRs.

We input the gene names shown in Table 3 to STRING 9.1 and searched for the known and predicated protein-protein interaction network. To reduce network complexity, only 10 more partners of strongest interaction evidence were added to the network of query genes.

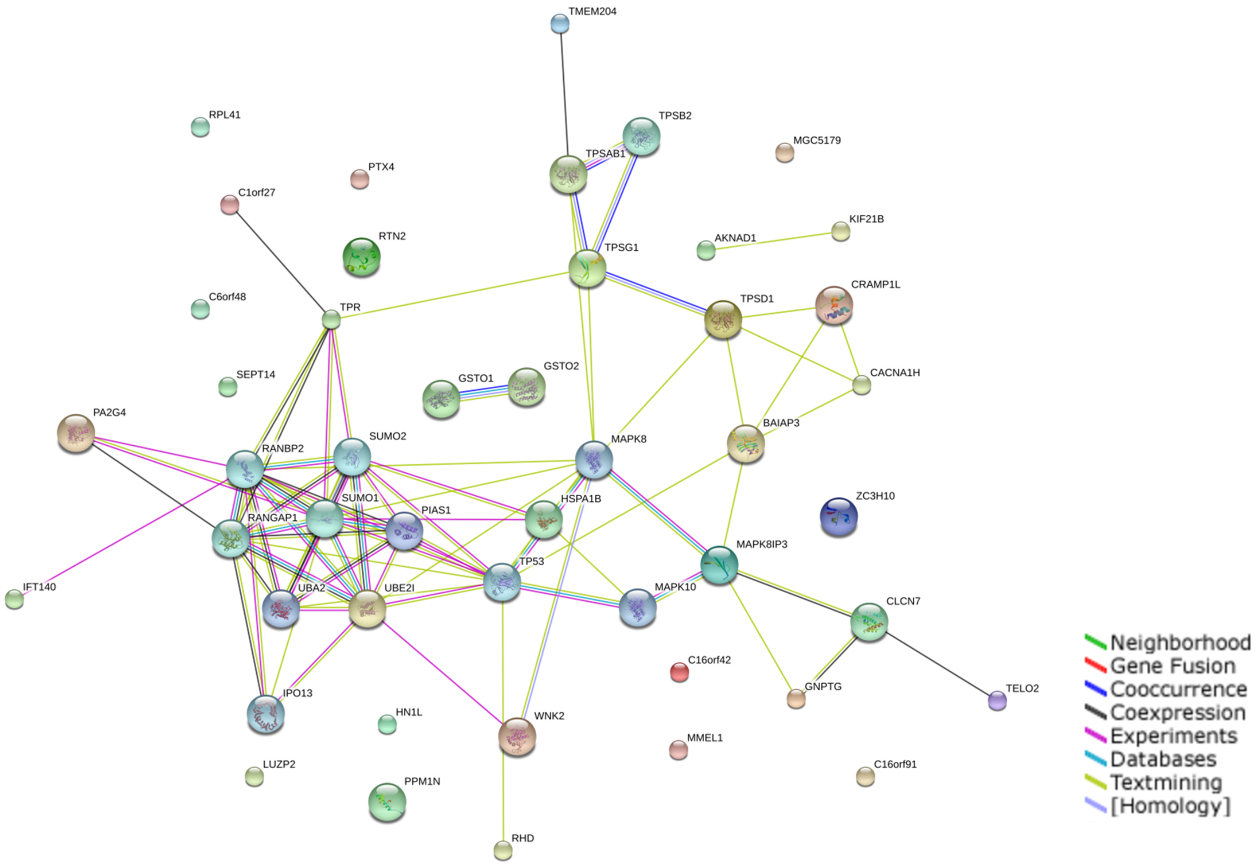

Supplementary Figure 6. Predicted functional coupling network of murine homologues of genes the exons of which overlap with significant CNVRs.

We input the gene names shown in Table 3 to FunCoup 3.0 and searched for the known and predicated functional coupling network among their murine homologues. To reduce network complexity, only 10 more partners of strongest interaction evidence were added to the network of query genes.

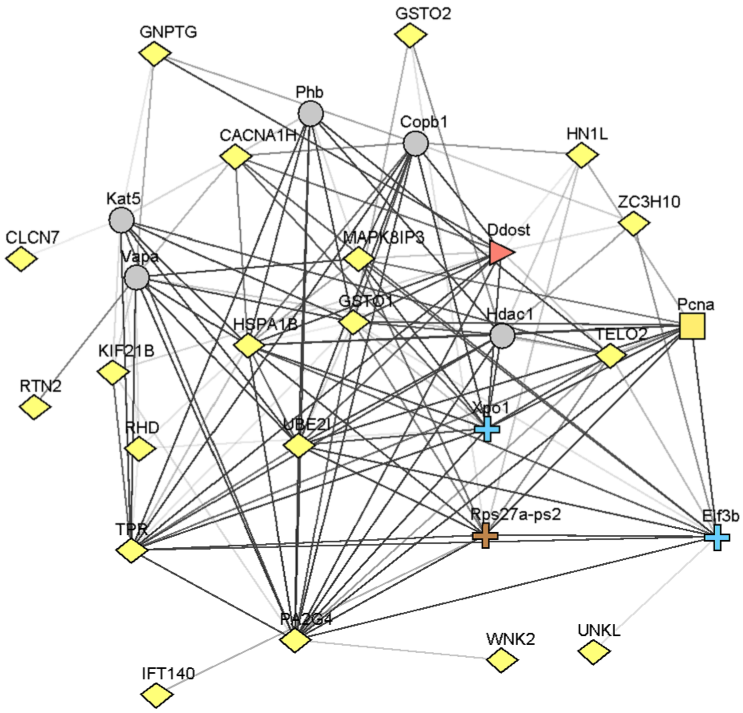

Supplementary Figure 7. Epigenetics profiles of the CNVR (chr1:109,367,944-109,371,874) in gene AKNAD1.
(a) histone markers in pancreatic islets; (b) histone markers in adult liver; (c) DNaseI hypersensitivity sites in pancreatic islets; (d) chromatin states in pancreatic islets and HepG liver tumor cell line (chromatin state keys are indicated at the bottom right corner).

a

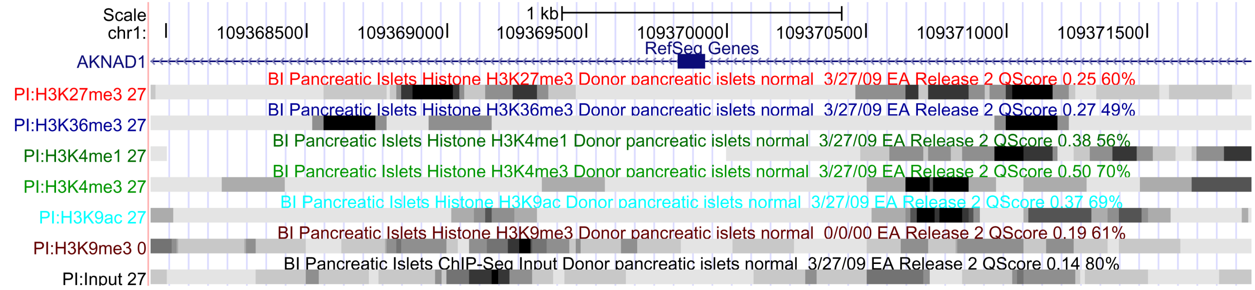

b

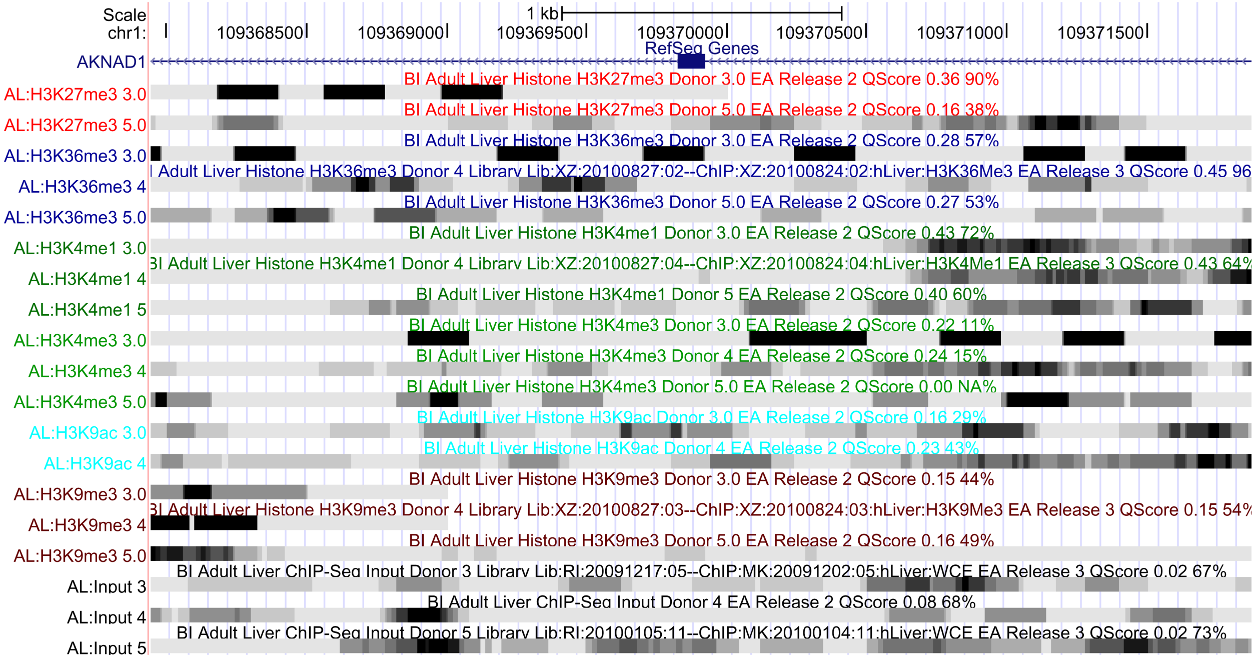

c

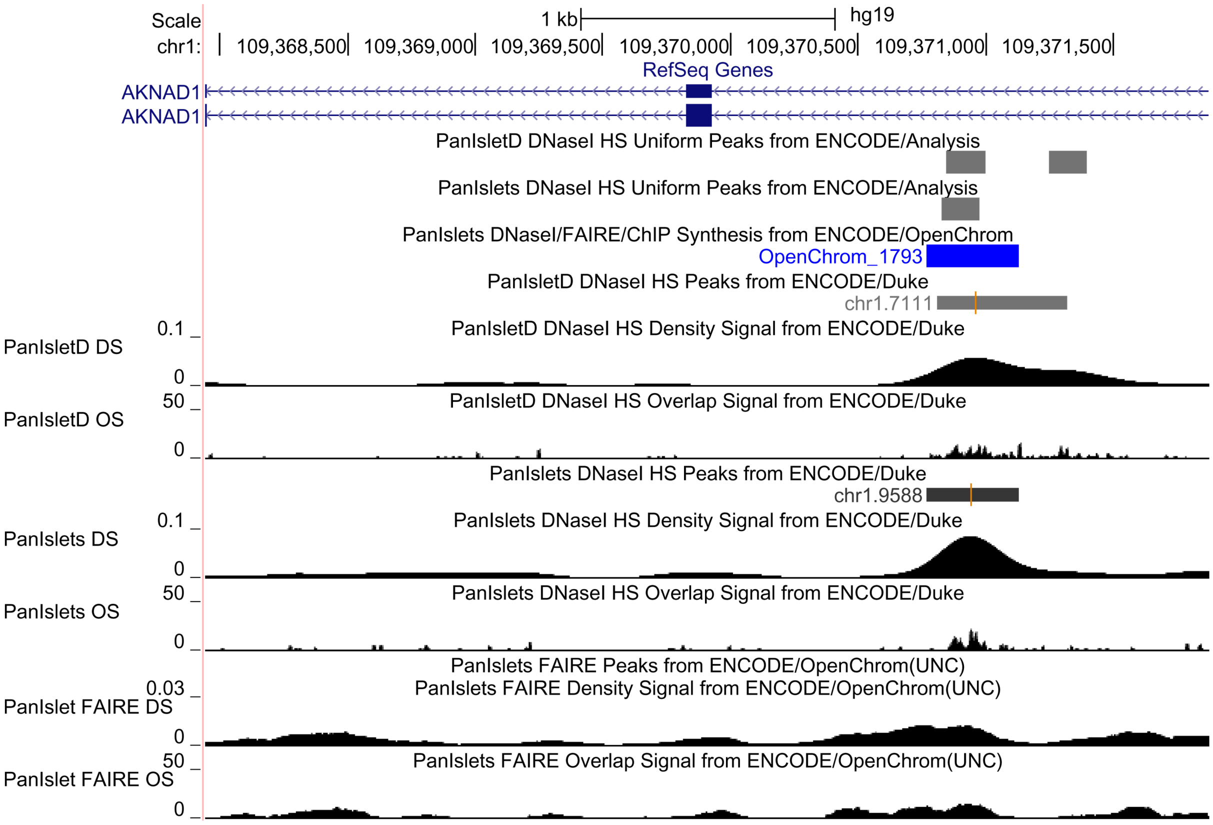
Scale
1 kb
hg19
chr1:
109,368,500
109,369,000
109,369,500
109,370,000
109,370,500
109,371,000
109,371,500
RefSeq Genes
AKNAD1
AKNAD1
PanIsletD DNaseI HS Uniform Peaks from ENCODE/Analysis
PanIslets DNaseI HS Uniform Peaks from ENCODE/Analysis
PanIslets DNaseI/FAIRE/ChIP Synthesis from ENCODE/OpenChrom
OpenChrom_1793
PanIsletD DNaseI HS Peaks from ENCODE/Duke
chr1.7111
0.1
PanIsletD DS
PanIsletD DNaseI HS Density Signal from ENCODE/Duke
0
50
PanIsletD OS
PanIsletD DNaseI HS Overlap Signal from ENCODE/Duke
0
PanIslets DNaseI HS Peaks from ENCODE/Duke
chr1.9588
0.1
PanIslets DS
PanIslets DNaseI HS Density Signal from ENCODE/Duke
0
50
PanIslets OS
PanIslets DNaseI HS Overlap Signal from ENCODE/Duke
0
PanIslets FAIRE Peaks from ENCODE/OpenChrom(UNC)
0.03
PanIslet FAIRE DS
PanIslets FAIRE Density Signal from ENCODE/OpenChrom(UNC)
0
50
PanIslet FAIRE OS
PanIslets FAIRE Overlap Signal from ENCODE/OpenChrom(UNC)
0

d

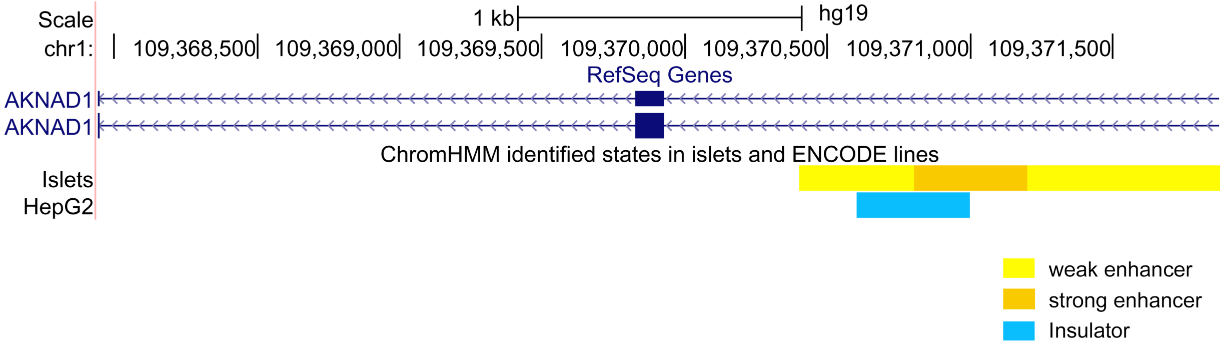
Scale
1 kb
hg19
chr1:
109,368,500
109,369,000
109,369,500
109,370,000
109,370,500
109,371,000
109,371,500
RefSeq Genes
AKNAD1
AKNAD1
ChromHMM identified states in islets and ENCODE lines
Islets
HepG2
weak enhancer
strong enhancer
Insulator

References

1 Snel, B., Lehmann, G., Bork, P. & Huynen, M. A. STRING: a web-server to retrieve and display the repeatedly occurring neighbourhood of a gene. *Nucleic Acids Res* **28**, 3442-3444 (2000).

2 Franceschini, A. *et al.* STRING v9.1: protein-protein interaction networks, with increased coverage and integration. *Nucleic Acids Res* **41**, D808-815, doi:10.1093/nar/gks1094 (2013).

3 Alexeyenko, A. & Sonnhammer, E. L. Global networks of functional coupling in eukaryotes from comprehensive data integration. *Genome Res* **19**, 1107-1116, doi:10.1101/gr.087528.108 (2009).

4 Schmitt, T., Ogris, C. & Sonnhammer, E. L. FunCoup 3.0: database of genome-wide functional coupling networks. *Nucleic Acids Res* **42**, D380-388, doi:10.1093/nar/gkt984 (2014).

5 Ward, L. D. & Kellis, M. HaploReg: a resource for exploring chromatin states, conservation, and regulatory motif alterations within sets of genetically linked variants. *Nucleic Acids Res* **40**, D930-934, doi:10.1093/nar/gkr917 (2012).

6 Su, A. I. *et al.* A gene atlas of the mouse and human protein-encoding transcriptomes. *Proc Natl Acad Sci U S A* **101**, 6062-6067, doi:10.1073/pnas.0400782101 (2004).

7 Wu, C. *et al.* BioGPS: an extensible and customizable portal for querying and organizing gene annotation resources. *Genome Biol* **10**, R130, doi:10.1186/gb-2009-10-11-r130 (2009).

8 Wu, C., Macleod, I. & Su, A. I. BioGPS and MyGene.info: organizing online, gene-centric information. *Nucleic Acids Res* **41**, D561-565, doi:10.1093/nar/gks1114 (2013).
